# Spontaneous splenic rupture in a patient with chronic myeloid leukemia: A case report

**DOI:** 10.1016/j.ijscr.2019.11.051

**Published:** 2019-11-30

**Authors:** Roberto Rueda-Esteban, Nicolás Stozitzky Muñoz, Mónica Barrios Díaz, Andrés García Sierra, Carlos Felipe Perdomo

**Affiliations:** a Universidad de los Andes School of Medicine, Carrera 1A No. 18A-10, Bogotá, Colombia; b Fundación Santa Fe de Bogotá, Carrera 7 No. 117 – 15, Bogotá, Colombia

**Keywords:** SSR, spontaneous splenic rupture, CML, chronic myeloid leukemia, AAST, American Association for the Surgery of Trauma, WSES, World Society of Emergency Surgery, Case report, Spontaneous rupture, Splenic rupture, Myeloid leukemia, Abdominal pain, Acute pain

## Abstract

**Introduction:**

Spontaneous splenic rupture is an atraumatic event that represents a rare and life-threatening acute complication in which the spleen is damaged producing internal hemorrhage in the abdominal cavity. Its association with hematologic malignancies, although a rare occurrence, has been previously described. Among this subset of patients, chronic myeloid leukemia is one of the main causes.

**Presentation of case:**

A 26-year-old male with history of chronic myeloid leukemia presented with acute intense abdominal right lower quadrant pain. Computed tomography showed a wedge in the lower third of the spleen (probably associated with infarction), active bleeding, and hemoperitoneum. Laparotomy and splenectomy were performed.

**Discussion:**

The most common symptom of spontaneous splenic rupture is acute abdominal pain, sometimes radiating to the left shoulder. It can also be associated with nausea, emesis and signs of hypovolemia or shock. Splenomegaly may be absent. Diagnostic methods of choice are computed tomography and ultrasound.

Management of splenic rupture is divided in surgical and conservative. The former is reserved for patients with extensive splenic injury that is accompanied by hemodynamic instability or other trauma that warrants surgical treatment. Patients who do not meet these criteria and respond to initial stabilization strategies can be offered clinical and laboratory monitoring. Stable patients with moderate to severe splenic injuries can be offered angioembolization.

**Conclusion:**

It is important to include splenic rupture as a differential diagnosis for acute abdominal pain, especially in patients with hematologic malignancy, since early recognition and treatment increases patient survival and improves prognosis.

## Highlights

• SSR is mainly caused by one of three conditions: hematological malignancies, infectious diseases, and other inflammatory or neoplastic disorders.
• CML is one of the main causes of SSR along with Hodgkin lymphoma.
• Three mechanisms are believed to cause SSR: parenchymal congestion and concomitant coagulopathy, leadingto splenic hemorrhage and infarction.
• In only 19 % of reported cases, SSR was diagnosed correctly; most common differential diagnoses include hepatic and biliary pathologies.
• Timely diagnosis and treatment improves survival rate and reduces complications.

## 1 Introduction

Splenic rupture is a rare and life-threatening acute complication in which the spleen is damaged producing internal hemorrhage in the abdominal cavity [1]. Spontaneous rupture is considered an atraumatic event [2]. It is a rare abdominal emergency that requires both immediate diagnosis and early treatment to ensure patient survival [3]. Spontaneous splenic rupture (SSR) was described for the first time in 1924 [4]. SSR secondary to hematologic malignancy is rare, but among this subset of patients, chronic myeloid leukemia (CML) is one of the main causes [5]. This is a case report of a spontaneous splenic rupture secondary to severe splenomegaly in a patient with CML. This clinical case is reported in line with the SCARE criteria [19].

## 2 Presentation of case

A 26-year-old male patient with history of CML was admitted to our hospital’s emergency department for acute abdominal pain. The pain was intense, had initiated 2 h before presentation, and was located in the right lower quadrant and right iliac region. Nausea without emesis and dysuria were also present. Diagnosis of CML had been made 2 months prior to the index event and he had rejected chemotherapy due to personal reasons; further, he denied taking other medications and there was no relevant family history. At admission, the patient had the following vital signs: heart rate 80 beats/min, respiratory rate 18 breaths/min, arterial pressure 123/71 mmHg, body temperature 36.0 °C, and oxygen saturation 87 % in ambient air. Physical examination was notable for mucocutaneous pallor and a distended abdomen; abdominal guarding and rebound tenderness were absent. The patient reported no history of trauma. Differential diagnoses initially considered included blast crisis, urolithiasis, pneumoperitoneum or abdominal viscera infarction.

After 2 h in the emergency department, the patient reported an increase in pain which was unresponsive to high doses of opioids.

**Fig. 1:**
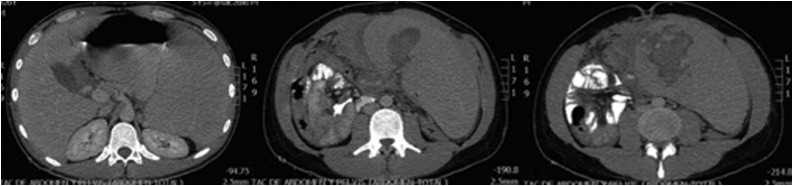
Abdominal CT scan, axial slices at T12 (left), L2 (middle) and L3-L4 intervertebral disc (right). Patient data was deleted.

**Fig. 2:**
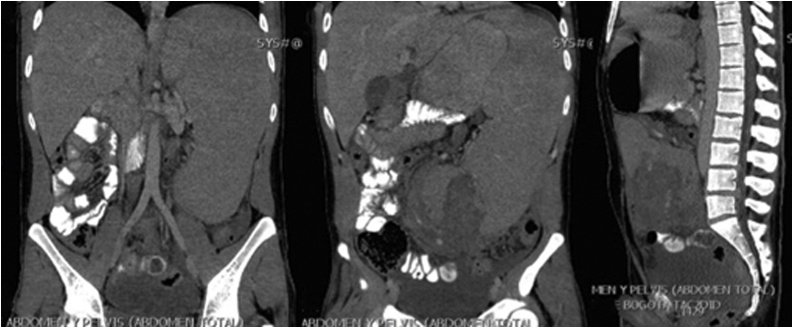
Abdominal CT scan coronal slices at abdominal aorta level (Left) Cecum (Middle) and midline sagittal slice (Right). Patient data was deleted.

**Fig. 3:**
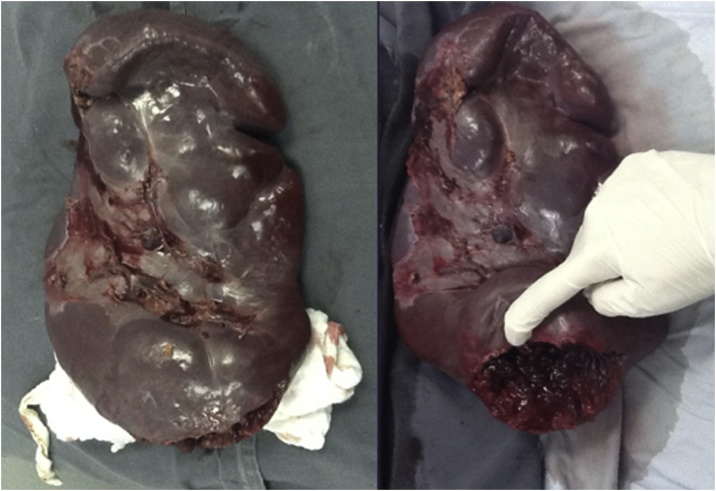
Surgical specimen (Spleen). Single surgical piece (Left). Surgical piece showing the site of the rupture (Right).

Initial workup included a normal chest radiograph and urinalysis, a complete blood count with evidence of leukocytosis (270.000/mm^3^), low hemoglobin (11.2 g/dL) and hematocrit (33.3 %) values, and thrombocytosis (925.000 platelets). Hypokalemia was also detected (2,96 mEq/L). Other blood tests were unremarkable (BUN 10 mg/dl, creatinine 0,8 mg/dl, sodium 136 mEq/L, chloride 100,6 mEq/L, INR 1.25). Abdominal computed tomography (CT) scan was also performed, which reported “important hepatosplenomegaly, an ill-defined lesion with irregular infiltrating edges and vascular tracts, and free fluid in the abdominal cavity” (axial and coronal images are presented in Fig. 1, Fig. 2, respectively). The lesion was initially considered compatible with neoplasia or spleen necrosis. A wedge was observed in the lower third of the spleen (probably associated with infarction), active bleeding, and hemoperitoneum. By the time the CT scan was evaluated by surgery staff, the patient’s clinical condition had deteriorated; he was pale and diaphoretic, with abdominal guarding and rebound tenderness. Vital signs showed tachycardia (134 beats per minute), tachypnea (30 per minute), hypertension (150/70 mmHg) and low oxygen saturation (84 %). Arterial blood gas analysis demonstrated metabolic acidosis (pH 7.0). Based on the suspicion of hypovolemic shock, surgical management was chosen, and an emergency exploratory laparotomy was performed by one of the hospital’s senior general surgeon. Surgical findings were hemoperitoneum of 1.5 L with active bleeding, numerous blood clots and splenic segments. A total splenectomy was required, obtaining a surgical specimen (spleen) of 2 kg and 32 cm of crown-rump length with an important rupture of 11 × 2.5 cm in the lower pole (Fig. 3).

During the procedure, the patient required transfusion of 6 units of red blood cells and administration of abundant crystalloids.

Microscopic examination of the specimen reported mature myeloid population infiltration reactive to CD15 in the red pulp. Additionally, larger cells with blast morphology and minimal compliance for CD117, and some cells with paranuclear positivity for CD34 and histiocytic appearance were reported. Inspection with the CD38 marker did not yield positive results. Other relevant findings included important lymphoid depletion and infarct changes near the zone of rupture. There was no evidence of bacteria or fungi.

The patient was initially transferred to the intensive care unit for monitoring. His postoperative evolution was satisfactory, specific treatment for CML was initiated and he was discharged 14 days after the surgical procedure.

## 3 Discussion

In most cases, SSR is caused by one of three main conditions: hematological malignancy, e.g. acute leukemia or lymphoma (30.3 %); infectious diseases, such as malaria, mononucleosis or other viral infections (27.3 %); and inflammatory or neoplastic disorders like acute or chronic pancreatitis (20.0 %). Other described causes include drugs and chemotherapy such as treatment with filgrastim (9.2 %) and mechanical disorders (6.8 %). A small number of cases have been described in patients with normal spleens and no underlying condition (6.4 %)5. SSR has an approximate 12 % mortality [6], but in patients with neoplastic disorders it can reach approximately 21 % [7]. Nevertheless, an early surgical treatment increases patient’s survival up to 60 % [8].

SSR secondary to hematological neoplasms is rare. According to the literature reviewed, approximately 200 cases have been reported; chronic myeloid leukemia appears to be one of the main causes (15.8 %) along with Hodgkin lymphoma (36.2 %). Other causes are myeloproliferative disorders (15.8 %), acute myeloid leukemia (13.8 %), acute lymphoblastic leukemia (7.9 %), and myelodysplastic disorders (7.9 %) [5].

Several mechanisms through which rupture occurs have been postulated. Among these the most important is congestion of splenic parenchyma and infiltration by blast cells. Nevertheless, concomitant coagulation disorders are likely present leading to sub-capsular hemorrhage and splenic infarction [3].

The most common symptom observed in SSR is acute abdominal pain of variable location and intensity, sometimes radiating to the left shoulder (Kehr's sign). It can also be associated with hypotension, fever, tachycardia, tachypnea, nausea, vomiting and low hematocrit values, reflecting active hemorrhage inside the abdominal cavity. Splenomegaly may be absent; however, this does not rule out the diagnosis [9,10]. Acute abdominal pain is the most frequent symptom of an SSR in patients with underlying hematologic malignancy (95 % of cases). This pathology should always be suspected in this clinical context, especially if the pain is accompanied by hypotension [2,[9].

Diagnostic methods of choice are CT scan and ultrasound. Nevertheless, only 19 % of SSR cases reported in the literature were diagnosed correctly [10]. Usually, the differential diagnosis of SSR includes acute hepatitis, biliary duct obstruction, intestinal perforation, acute pancreatitis and angina [11,15]; distinguishing between these entities on clinical grounds can often be challenging.

In this patient, a grade IV splenic rupture was found, according to the spleen damage scale of the American Association for the Surgery of Trauma (AAST) [12], indicating a laceration of segmental or hilar vessels that produces devascularization of over 25 % of the parenchyma. If the World Society of Emergency Surgery (WSES) classification is used the patient would be said to have a severe spleen injury (class IV) due to the development of hemodynamic instability [16]. These findings rendered him not eligible for non-operative management, thus splenectomy was promptly carried out.

Management of splenic rupture has been classically divided into surgical (splenectomy) and conservative (clinical monitoring). According to the 2017 splenic trauma guidelines, the former is reserved for patients with extensive splenic injury that is often accompanied by hemodynamic instability or other concurrent trauma that warrants surgical treatment. This means that patients who do not meet these criteria and respond to initial stabilization strategies can be offered close clinical and laboratory monitoring. Nevertheless, another group of patients has been recently identified that would benefit from angioembolization techniques. Such patients typically are hemodynamically stable at the time of procedure, have moderate to severe splenic injuries and show associated vascular abnormalities on CT scan. It has also been proposed as a salvage therapy for monitored patients that show signs of persistent hemorrhage. In any case, if angioembolization fails to control hemorrhage surgical treatment must be offered [16].

Splenectomy has a morbidity rate of 14–61% and a mortality rate of 0–28%. These values are presumed to be higher in patients with hematologic malignancies due to an increased risk of bleeding and infection. The most commonly associated risk factors are spleen weight over 2 kg, male gender and increased surgical time. Moreover, a variety of postoperative complications exist, such as subphrenic abscess, hemoperitoneum, respiratory complications, postsplenectomy fever, sepsis, thrombocytosis, among others. Splenectomy performed by advanced laparoscopic surgery reduces the incidence of these complications but is usually not recommended in the trauma setting [3,13,14,16].

Other surgical approaches (e.g. partial splenectomy) are usually recommended for localized diseases of the spleen, which is not the case for most causes of SSR [17,18].

Importantly, current evidence-based guidelines have been proposed for the management of splenic traumatic injury. On the contrary, SSR remains a somewhat uncommon pathology and its management is largely based on local experience, case reports and extrapolated data from trauma cases.

## 4 Conclusion

SSR is an important differential diagnosis for acute abdominal pain, especially in patients with underlying hematologic malignancy. Timely diagnosis and treatment is associated with a high survival rate and less complications.

## Sources of funding

The study had no sponsors and no funding was required.

## Ethical approval

In our institution case reports do not require ethical committee approval.

## Consent

Written informed consent was obtained from the patient for publication of this case report and accompanying images

## Author contribution

**Roberto Rueda-Esteban:** Conceptualization, Methodology, Investigation, Writing – Review & Editing, Visualization, Supervision**. Nicolás Stozitzky Muñoz:** Investigation, Writing – Original Draft, Writing – Review & Editing, Visualization**. Mónica Barrios Díaz:** Investigation, Writing – Original Draft, Visualization. **Andrés García Sierra:** Investigation, Writing – Original Draft, Visualization. **Carlos Felipe Perdomo:** Conceptualization, Methodology, Writing – Review & Editing, Supervision.

## Registration of research studies

This case report does not require registration.

## Guarantor

Roberto Rueda-Esteban

## Provenance and peer review

Not commissioned, externally peer-reviewed

## Declaration of Competing Interest

Nothing to declare.
